# Familial vs. sporadic sarcoidosis: BTNL2 polymorphisms, clinical presentations, and outcomes in a French cohort

**DOI:** 10.1186/s13023-016-0546-4

**Published:** 2016-12-03

**Authors:** Yves Pacheco, Alain Calender, Dominique Israël-Biet, Pascal Roy, Serge Lebecque, Vincent Cottin, Diane Bouvry, Hilario Nunes, Pascal Sève, Laurent Pérard, Gilles Devouassoux, Nathalie Freymond, Chahira Khouatra, Benoît Wallaert, Raphaelle Lamy, Mad-Hélénie Elsensohn, Claire Bardel, Dominique Valeyre, GSF group, A. Gouita, P. Chanez, E. Peyrat-Detis, G. Prevot, S. Diab, F. Prieur, M. Locatelli-Sanchez, M. Gaillot-Drevon, L. Kiakouama, C. Broussolle, L. Varron, A. Charron, J. Kuntz, K. Ledoux, M. C. Le Scanff, Bouaziz, M. Pavic, J. Ninet, J. F. Cordier, M. Vincent, M. Coudurier, F. Gagnadoux, F. Jounieaux, G. Terce, P. Diot, S. Marchand-Adams, H. Morel, S. Jouneau, L. Olivier-Faivre, J. Lacronique, K. Juvin, B. Crestani, M. Aubier, R. Borie, R. Roussel, C. Chapelon Abric, B. Hervier, J. C. Piette, T. Janssens, D. Saadoun, N. Nathan, A. Clément, J. P. Ducroix, C. Guillaumat, E. Watkin, M. Freynet, J. M. Naccache, Y. Uzunhan, N. Limal

**Affiliations:** 1 Hospices Civils de Lyon, Centre Hospitalier Lyon Sud, Université Claude Bernard – Lyon 1, EA-7426, Lyon, France; 2 Hospices Civils de Lyon, Hôpital Edouard Herriot, Plateforme de génétique moléculaire, CR-21076, Université Claude Bernard - Lyon 1, INSERM U1052, Lyon, France; 3 AP-HP, Hôpital Européen Georges Pompidou, Service de Pneumologie, Centre de Compétence des maladies pulmonaires rares, Université René Descartes – Paris 5, Paris, France; 4 Hospices Civils de Lyon, Service de Biostatistique, Université Claude Bernard – Lyon 1, CNRS UMR 5558, Lyon, France; 5 Hospices Civils de Lyon, Hôpital Lyon-Sud, Université Claude Bernard - Lyon1, INSERM U1052 - CNRS UMR5286, Lyon, France; 6 Hospices Civils de Lyon, Hôpital Louis Pradel, Université Claude Bernard - Lyon 1, UMR 754, Lyon, France; 7 AP-HP, Hôpital Avicenne, Université Paris13, EA2363, COMUE Sorbonne Paris Cité, Paris, France; 8 Hospices Civils de Lyon, Hôpital Croix-Rousse, Université Claude Bernard - Lyon 1, Lyon, France; 9 Hospices Civils de Lyon, Hôpital Edouard Herriot, Université Claude Bernard - Lyon 1, Lyon, France; 10 Centre hospitalo-universitaire de Lille, Service de Pneumologie et Immunoallergologie, Centre de Compétence Maladies Pulmonaires Rares, Université Lille 2, Lille, France; 11 Université Claude Bernard Lyon 1 - EA-7426, 165 Chemin du Grand Revoyet, F-69495 Pierre Benite, France

**Keywords:** Sarcoidosis, Medical genetics, Human BTNL2 protein, Candidate gene association study, Classification

## Abstract

**Background:**

The occurrence of familial forms of sarcoidosis (OMIM 181100) suggests a genetic predisposition. The involvement of butyrophilin-like 2 (BTNL2) gene (rs2076530 variant) has to be investigated.

**Results:**

The study performed independent analyses of BTNL2 polymorphism, clinical phenotypes, and outcomes in familial vs. sporadic presentations in 256 sporadic and 207 familial cases from 140 families. The logistic multivariate model showed that a young age at diagnosis and the combination of lung and skin involvement at diagnosis may distinguish sporadic from familial sarcoidosis (*p* = 0.016 and *p* = 0.041). We observed also that Sarcoid Clinical Activity Classification (SCAC) profiles were significantly different between familial and sporadic cases (*p* = 0.0497).

Variant rs2076530 was more frequent in patients than in controls (OR = 2.02; 95% CI: [1.32–3.09]) but showed no difference between sporadic and familial cases and no difference according to the clinical phenotype or the outcome.

**Conclusion:**

Despite a significant difference in BTNL2 polymorphism between sarcoid patients and controls, there was no such difference between familial and sporadic sarcoidosis cases and no correlation between BTNL2 polymorphism and disease severity or outcome. Thus, BTNL2 difference cannot be considered as a key marker for disease classification or patient management.

## Background

Sarcoidosis is a rare multisystemic granulomatous disorder of still unknown origin. It has various presentations, severities, treatments, and outcomes [1]. Genetic studies have searched for polymorphisms associated with the risk of developing the disease and for genotype-phenotype correlations. Among several candidate genes, BTNL2 (butyrophylin-like 2) has been intensively studied. A recent meta-analysis has confirmed that BTNL2 rs2076530 polymorphism contributes to the risk of sarcoidosis [2]. Meanwhile, it has been difficult to investigate the clinical phenotypes and outcomes of sarcoidosis in search for genotype-phenotype correlations. Nevertheless, interesting suggestions about classical radiographic staging [3], factor analysis [4, 5], SCAC (Sarcoid Clinical Activity Classification) [6], and WASOG (World Association for Sarcoidosis and Other Granulomatous Disorders) clinical outcome status classification [7] are available.

We investigated BTNL2 gene in familial forms of sarcoidosis to assess the role of this gene as a major indicator of hereditary predisposition to the disease [8–10] and find out whether it can be a useful genetic marker for clinical management and prognosis.

## Methods

### Participant inclusion

SARCFAM (a national project on familial sarcoidosis) is a prospective observational cohort study started in 2008. It involves 28 French university departments of internal medicine or pulmonology that care for sarcoidosis patients.

Three inclusion criteria were: i) clinical and paraclinical features consistent with sarcoidosis; ii) histopathological evidence of non-caseating granuloma, except Löfgren syndrome; and, iii) exclusion of any other chronic disease.

The evidence for non-caseating granulomatous lesions was obtained by mediastinoscopy (26.4%), or bronchial/pulmonary (34.9%), accessory salivary gland (12.3%), peripheral lymph node (10.4%), skin (9.8%), conjunctiva (2.5%), kidney (1.2%) or other (2.5%) biopsies.

All 463 cases included were diagnosed according to the Joint Statement of the American Thoracic Society (ATS), the European Respiratory Society (ERS), and the (WASOG) [11]. The disease type (familial vs. sporadic) was determined by structured interview.

The control population for the genetic investigation consisted of 430 DNA samples from a single genetic database of healthy human reference population. The mean age (41.5 ± 17.5 years) and geographic origins [European: *n* = 324 (75.3%) and Sub-Saharan African plus Caribbean: *n* = 106 (24.7%)] were quite similar to those of the study sarcoid patients.

### Clinical assessment

All patients with sporadic sarcoidosis or index cases with familial sarcoidosis were diagnosed and followed-up in the 28 clinical centers. A specific case report form was filled in with demographic data, medical history, disease onset (symptomatic or asymptomatic), detailed organ involvement, treatments, and outcome. A detailed familial history of sarcoidosis was also examined to assess genetic predisposition. Familial cases were offered a DNA sampling and the related forms were filled in the center where the index case was diagnosed.

Clinical, biological, and imaging data were also collected at diagnosis and each follow-up visit until December 2012. These data included: i) chest X-ray staging (0: no involvement, 1: isolated hilar lymphadenopathy, 2: hilar lymphadenopathy with pulmonary infiltration, 3: isolated pulmonary infiltration without fibrosis, 4: pulmonary fibrosis); ii) pulmonary function testing: forced expiratory volume in one second (FEV1), forced vital capacity (FVC), FEV1/FVC ratio, and total lung capacity (TLC); iii) biological variables: serum calcium and creatinine, bronchoalveolar lavage cell count; and, iv) information about treatment: systemic treatment with corticosteroids or non-steroid immunosuppressive agents.

The disease outcome was examined using SCAC with six progression patterns [6] and, whenever possible, a classification of outcome in four categories: 1) recovery within 3 years; 2) recovery between 3 and 5 years; 3) no recovery at 5 years; 4) death.

### Genetic study

Blood DNAs were obtained from nearly all patients and tested for BTNL2 gene *rs2076530* polymorphism. Primer oligonucleotides for polymerase chain reaction and DNA sequencing were located within the reference sequence surrounding *rs2076530*, the major single-nucleotide polymorphisms (SNPs) that induce truncation of the BTNL2 protein [8]. A 490-bp amplicon was produced and sequenced using primers 5′-AATGCACAGAGCATGGAGGTGAG-3′ and 5′-GAAGATACTGGAAAAGATACAAG-3′.

The quality control of PCR products was performed on a LabChipGX system (PERKIN ELMER™) and the products purified by NucleoFast® 96 PCR Clean-up Kit from MACHEREY-NAGEL™. The sequencing was performed by Big Dye terminator v1.1 after purification with BigDye® XTerminator™ Purification Kit. Sequence delineation and base calling used an automated fluorescent DNA sequencer (Applied Biosystems™, model 3130xl).

### Statistical analysis

Familial and sporadic cases were compared using a generalized linear mixed model. To allow for family links, the model included correlations between patients’ random effects [12].

In a first application of the model, univariate submodels were constructed and compared with a null model (model with intercept only) using a likelihood ratio test to test the effects of the main variables (Table 1). The clinical characteristics of BTNL2 G/G vs. BTNL2 A/A plus BTNL2 A/G genotypes were compared with the same method (Table 2). Because the study was mainly exploratory, only raw *p*-values are shown; however, a Bonferroni correction for multiple testing was applied and when raw *p*-values were significant, adjusted *p*-values were also calculated [13].

**Table 1:** Epidemiological, clinical, and biological characteristics of familial and sporadic sarcoid patients

Data	Number	Familial cases	Sporadic cases	Univariate model raw *p* value*	Multivariate model *p* value
Epidemiological data					
N	463	207 (44.7%)	256 (55.3%)		
Sex	463			0.49	
Male	201	86 (41.6%)	115 (44.9%)		0.20
Female	262	121 (58.4%)	141 (55.1%)		Ref.
Age at diagnosis	424			0.001 [0.021]	0.016
Mean ± SD		38.2 ± 13.1	43.0 ± 15.5		
Median [q1; q3]		35.5 [29.3; 46.2]	40.3 [30.6; 54.0]		
Ethnicity	303			0.85	
European	235	112 (77.2%)	123 (77.9%)		
Caribbean or Sub-Saharan African	68	33 (22.8%)	35 (22.1%)		
Smokers	381			0.43	
Yes	156	56 (38.4%)	100 (42.6%)		
No	225	90 (61.6%)	135 (57.4%)		
Clinical data at diagnosis					
Disease onset	401			0.015 [0.28]	
Asymptomatic	137	45 (27.3%)	92 (39.0%)		
Symptomatic	264	120 (72.7%)	144 (61.0%)		
Löfgren syndrome	378			0.26	
Yes	58	29 (17.8%)	29 (13.5%)		
No	320	134 (82.2%)	186 (86.5%)		
Number of organs involved	414			0.144	
1	158	69 (39.9%)	89 (36.9%)		
2	117	36 (20.8%)	81 (33.6%)		
3	70	29 (16.8%)	41 (17.0%)		
≥ 4	69	39 (22.5%)	30 (12.5%)		
Organs involved	414			<0.001 [0.003]	
Lungs only	148	68 (39.31%)	80 (33.2%)		0.65
Lungs and Eyes	53	10 (5.78%)	43 (17.84%)		0.076
Lungs and Skin	21	15 (8.67%)	6 (2.49%)		0.041
Other organs	192	80 (46.24%)	112 (46.47%)		Ref
X-ray staging	456			0.20	0.78
0	68	40 (19.8%)	28 (11.0%)		
1	162	66 (32.7%)	96 (37.8%)		
2	157	69 (34.2%)	88 (34.7%)		
3	48	15 (7.4%)	33 (13.0%)		
4	21	12 (5.9%)	9 (3.5%)		
PFT at first visit					
TLC (%)	215			0.93	
< 80%	47	18 (22.2%)	29 (21.6%)		
≥ 80%	168	63 (77.8%)	105 (78.4%)		
FEV1/FVC	242			0.43	
< 70%	55	18 (20.0%)	37 (24.3%)		
≥ 70%	187	72 (80.0%)	115 (75.7%)		
Biology at first visit					
Bronchoalveolar lavage lymphocytes (%)	95			0.171	
< 10	23	3 (11.6%)	20 (29.0%)		
[10; 20]	17	5 (19.2%)	12 (17.4%)		
> 20	55	18 (69.2%)	37 (53.6%)		
Calcemia	256			0.80	
< 2.25	52	21 (22.6%)	31 (19.0%)		
[2.25 - 2.5]	182	64 (68.8%)	118 (72.4%)		
> 2.5	22	8 (8.6%)	14 (8.6%)		
Treatment					
Treatment	463			<0.001 [0.008]	
None	136	79 (38.2%)	57 (22.3%)		
At least one	327	128 (61.8%)	199 (77.7%)		
Type of treatment	327			0.188	
Corticosteroids only	186	67 (52.3%)	119 (59.8%)		
Other treatment	141	61 (47.7%)	80 (40.2%)		
Pattern of disease course					
SCAC	414			0.003 [0.0497]	
1- symptomatic - no treatment	48	30 (18.1%)	18 (7.3%)		
2- symptomatic - treatment ≤ 12 mo.	47	18 (10.8%)	29 (11.7%)		
3- symptomatic - treatment > 12 mo.	172	72 (43.4%)	100 (40.3%)		
4- asymptomatic - no treatment	51	19 (11.5%)	32 (12.9%)		
5- asymptomatic - treatment ≤ 12 mo.	16	7 (4.2%)	9 (3.6%)		
6- asymptomatic - treatment > 12 mo.	80	20 (12.0%)	60 (24.2%)		
Classification of outcome	138			0.165**	
1- recovery within 3 years	72	24 (57.14%)	48 (50%)		
2- recovery between 3 and 5 years	32	6 (14.29%)	26 (27.08%)		
3- no recovery at 5 years	30	12 (28.57%)	18 (18.75%)		
4- death	4	0 (0%)	4 (4.17%)		
Genetic data					
BTNL2	436			0.17***	0.39***
BTNL2 A/A	201	89 (47.8%)	112 (44.8%)		
BTNL2 A/G	194	84 (45.2%)	110 (44.0%)		
BTNL2 G/G	41	13 (7.0%)	28 (11.2%)		

*Significant raw *p*-values are followed by corrected *p*-values (Bonferroni correction) in brackets

**Small “Death” category excluded

***G/G vs. G/A + A/A

**Table 2:** Comparison of clinical characteristics according to the BTLN2 genotype (G/G vs. A/A plus G/A)

Data	Number	BTNL2			Univariate model raw *p* value *,**
		A/A	G/A	G/G	
Epidemiological data					0.59
Ethnicity	292				
European	226	103 (75.7%)	104 (80%)	19 (73.1%)	
Caribbean or Sub-Saharan African	66	33 (24.3%)	26 (20%)	7 (26.9%)	
Clinical data at diagnosis					
Disease onset	389				0.91
Asymptomatic	130	60 (34.29%)	59 (33.15%)	11 (30.56%)	
Symptomatic	259	115 (65.71%)	119 (66.85%)	25 (69.44%)	
Löfgren syndrome	368				0.042 [0.42]
Yes	58	25 (14.71%)	32 (19.39%)	1 (3.03%)	
No	310	145 (85.29%)	133 (80.61%)	32 (96.97%)	
Number of organs involved	397				0.074
1	152	70 (38.46%)	73 (41.01%)	9 (24.32%)	
2	115	55 (30.22%)	49 (27.53%)	11 (29.73%)	
3	66	29 (15.93%)	33 (18.54%)	4 (10.81%)	
≥ 4	64	28 (15.39%)	23 (12.92%)	13 (35.14%)	
Organs involved	397				0.082
Lungs only	143	68 (37.36%)	67 (37.64%)	8 (21.62%)	
Lungs and Eyes	52	23 (12.64%)	20 (11.24%)	9 (24.33%)	
Lungs and Skin	20	11 (6.04%)	8 (4.49%)	1 (2.7%)	
Other sites	182	80 (43.96%)	83 (46.63%)	19 (51.35%)	
X-ray staging	432				0.26
0	57	25 (12.5%)	26 (13.47%)	6 (15.38%)	
1	158	77 (38.5%)	65 (33.68%)	16 (41.03%)	
2	149	67 (33.5%)	68 (35.23%)	14 (35.9%)	
3	47	24 (12%)	22 (11.4%)	1 (2.56%)	
4	21	7 (3.5%)	12 (6.22%)	2 (5.13%)	
PFT at first visit					
TLC (%)					0.098
< 80%	47	23 (24.47%)	22 (23.91%)	2 (9.52%)	
≥ 80%	160	71 (75.53%)	70 (76.09%)	19 (90.48%)	
Pattern of disease course					
SCAC	398				0.83
1- symptomatic - no treatment	46	21 (11.6%)	22 (12.1%)	3 (8.3%)	
2- symptomatic - treatment ≤ 12 mo.	47	19 (10.5%)	22 (12.1%)	6 (16.7%)	
3- symptomatic - treatment > 12 mo.	166	75 (41.4%)	74 (40.9%)	17 (47.2%)	
4- asymptomatic - no treatment	48	19 (10.5%)	26 (14.4%)	3 (8.3%)	
5- asymptomatic - treatment ≤ 12 mo.	14	6 (3.3%)	7 (3.9%)	1 (2.8%)	
6- asymptomatic - treatment > 12 mo.	77	41 (22.7%)	30 (16.6%)	6 (16.7%)	
Classification of outcome	137				0.015 [0.14]***
1- recovery within 3 years	72	29 (52.73%)	36 (52.17%)	7 (53.85%)	
2- recovery between 3 and 5 years	32	9 (16.36%)	17 (24.64%)	6 (46.15%)	
3- no recovery at 5 years	29	15 (27.27%)	14 (20.29%)	0 (0%)	
4- death	4	2 (3.64%)	2 (2.9%)	0 (0%)	

*Significant raw *p*-values are followed by corrected *p*-values (Bonferroni correction) in brackets

**Small “Death” category excluded

***G/G vs. G/A + A/A

In another application, a multivariate model was built with the most important variables collected at diagnosis (age, sex, organs involved, chest X-ray stage, and BTNL2 polymorphism) to test their association with familial cases.

## Results

### The study population

Among the 463 patients, 256 presented with a sporadic disease and 207 belonged to 140 families (one patient from each of 85 families, two patients from each of 48 families, three patients from each of five families, four patients from one family, and seven patients from another one). Most of the 303 patients with known ancestors’ birth countries were European (78%); the others were Caribbean or Sub-Saharan African (22%). This distribution did not differ from that of the controls. Table 1 shows a comparison of patient epidemiological, clinical, and biological characteristics between sporadic and familial cases.

### Impact of disease type

Univariate comparisons between sporadic and familial cases revealed no significant differences in terms of sex ratio, geographical origin, number of involved organs at diagnosis, distribution of chest X-ray stages at diagnosis, respiratory function tests, biological data, or smoking habits. Also, the incidence of Löfgren syndrome was not different between familial and sporadic cases (17.8% vs. 13.5%, respectively). However, the age at diagnosis was lower in familial cases (*p* = 0.021 after Bonferroni correction) and the combinations of organs involved at diagnosis were significantly different (*p* = 0.003 after Bonferroni correction).

Sporadic cases needed treatment more often than familial cases (*p* = 0.008 after Bonferroni correction). Among treated patients, 52.3% of familial and 59.8% of sporadic cases received exclusively corticosteroids. Other treatments (methotrexate, azathioprine, aminoquinolines, or anti-TNF alpha) were given to 47.7% of familial and 40.2% of sporadic cases. The SCAC was significantly different between familial and sporadic cases (*p* = 0.0497 after Bonferroni correction) but the classification of outcome did not show a significant difference.

A multivariate model was then constructed with the following variables assessed at diagnosis: age, sex, chest X-ray stage, organs involved, and BTNL2 polymorphism. The model was run on the 390 patients without missing data (233 sporadic and 157 familial cases, the former being the reference). The OR estimates and their 95% CIs were 0.81 [0.69; 0.96] for age (per ten-year increment) and 0.73 [0.45; 1.18] for sex (males vs. females). Considering an additive effect for ordinal variables and a dominant genetic model for BTNL2 polymorphism (GG being the wild-type homozygous genotype), the OR estimates were: 1.04 [0.80; 1.34] for chest X-ray stage (per stage increment), 1.46 [0.62; 3.45] for BTNL2 (G/G vs. G/A plus A/A), 1.13 [0.67; 1.88] for organs involved “lungs only”, 0.47 [0.21; 1.08] for “lungs and eyes”, 3.16 [1.05; 9.54] for “lungs and skin” (“other organs” being the reference). In agreement with the univariate analyses, familial cases were significantly younger at diagnosis than sporadic ones (*p* = 0.016) and the organs involved in familial cases were more often “lungs and skin” (*p* = 0.041).

### Impact of BTNL2

In the control population (*n* = 430), the genotypes observed for SNP rs2076530 (normal allele vs. mutated variant A) were respectively 75 (17.4%) G/G, 143 (33.2%) G/A, and 212 (49.3%) A/A. The relative distribution of genotype G/G vs. G/A plus A/A was significantly different between sarcoidosis cases and the control population (odds ratio, OR = 2.02; 95% confidence interval, CI: [1.32–3.09]). The same comparison performed between familial and sporadic cases showed no statistically significant difference.

Table 2 shows that, in the whole population of patients, there is no correlation between BTNL2 polymorphism and the various clinical phenotypes, the SCAC, or the classification of outcome.

## Discussion

The present study was based on clinical and genetic investigations in a nation-wide cohort of both familial and sporadic sarcoidosis cases. Comparisons between familial and sporadic cases showed some clinical differences but, mostly, the absence of difference in BTNL2 rs2076530 variant frequency. Moreover, the study found no genotype-phenotype correlation between BTNL2 and clinical phenotype, SCAC, or the classification of outcome.

Familial aggregation of sarcoidosis cases is already well known [14]; it raised the issues of predisposing genes and genotypes involved in a Mendelian inheritance of the clinical phenotype. Along with ACCESS [15], we assessed the clinical phenotypes and the outcomes in a large cohort of sarcoid patients comparing sporadic and familial cases. In agreement with the literature [16–19], we observed no difference in sex ratio or geographic origin between familial and sporadic cases. However, in contrast with other reports [16, 18, 19], age at diagnosis was significantly lower in the present study familial cases. This observation suggests that diagnosing sarcoidosis in individuals closely related to index cases in predisposed families may be made earlier than in sporadic cases. A previous work [17] compared the clinical profiles of patients whose diagnosis was made incidentally with symptomatic cases but could not conclude because of small sample sizes. In agreement with Sharma et al. [6], we found a difference between familial and sporadic cases regarding the disease onset (symptomatic vs. asymptomatic); this difference was statistically significant as per the raw *p* value but not significant after Bonferroni correction.

In addition, in agreement with other studies [16, 17], we found no differences in terms of chest X-ray staging or number of organs involved at diagnosis between familial and sporadic cases but the combinations of organs involved at diagnosis was significantly different between the two groups in univariate analyses. Also, the multivariate analysis showed a significant difference between familial and sporadic cases regarding lungs and skin combination. According to Delaveri et al. [20] such an observation may be in relation with the genetic predisposing background. Finally, as also reported by Sharma et al. [16], familial and sporadic cases did not differ in terms of drugs used (corticosteroids alone vs. immunosuppressive drugs).

Several investigators have designed comprehensive scoring systems to assess the disease impact and outcome [3–8]. Two of them have integrated the need for more than a 12-month treatment period [4, 6]. In the present study, we found a significant difference between familial and sporadic cases in terms of disease progression patterns (as per the SCAC) [6] but only a trend toward a difference in the classification of outcome classification according to the duration of the disease. This may reflect a lack of power regarding the classification of outcome. Indeed, only 138 patients had a sufficient follow-up to be correctly classified, whereas 344 patients could be classified with SCAC. A retrospective study of patients with an isolated lung involvement and 6–144 months of follow-up [5] has identified four disease phenotypes. We cannot easily refer to this series because of the predominance, in our series, of a multi-organ involvement.

The BTNL2-related OR we found here is similar to previously reported ones [2, 8–10]. However, our study adds the original findings that although BTNL2 polymorphism was strongly associated with the disease, its presence was not predictive of the development of a rather familial or rather sporadic case and not associated with the clinical phenotypes, the SCAC, or the outcome. This result differs from others that suggested that BTNL2 might be a predisposing factor for persistent or progressive sarcoidosis [10, 21, 22]. Our data may be interpreted in the light of the low OR of BTNL2 rs2076530 (=2) we found; that is, the BTNL2 gene is not an explanatory factor of disease inheritance. The role of the rs2076530 variant is probably part of a set of genetic and environmental factors associated with the risk of acquiring the disease.

Combined effects of HLA-DRB1/DBP1 haplotypes and the BTNL2 variant have been previously reported [23, 24]. Here, we cannot exclude that the BTNL2-sarcoidosis association we observed is the consequence of gene proximity in the 6p21 area with a synergistic effect of some HLA class II haplotypes and the truncated form of BTNL2 on T-cell activation and proliferation process [22, 25]. Actually, one limitation of the present study is the lack of extensive screening for HLA-DRB1/DPB1 in the patients. In familial cases, we have checked for SNPs within the HLA-DRB1/DPB1 genes in 9 unrelated index cases of familial sarcoidosis and found no common genotypes; this suggests that these index cases do not have a common ancestor and that there is no founder effect. We have also screened the full sequence of BTNL2 in a subset of 40 patients from 40 families and found no other variants than those described in the last version of the NCBI dbSNP database. Actually, we analysed the BTNL2 gene for other variants and found that the rs2076530 A/A variant genotype cosegregates with the rs2076520 synonymous V313V, rs28362679 missense S334L, and rs41441651 missense D336N polymorphisms and, to date, we cannot exclude that these polymorphisms may have a complementary role with that of rs2076530 in the malfunction of BTNL2. Taken together these data demonstrate clearly that, with an OR = 2.0, the rs2076530 splicing variant of BTNL2 should be considered as a genetic risk factor for sarcoidosis but cannot be considered as a major gene explaining a Mendelian inheritance in the familial form of the disease. As others, we suggest strongly that BTNL2 screening belongs to the panel of biomarkers in the diagnosis of sarcoidosis together with HLA class II haplotyping and further coming genes. Despite conflicting opinions, the 6p21.3 region carrying HLA and BTNL2 genes is still considered to be associated with an increased risk of TH1/TH17 diseases (tuberculosis, leprosy, beryllium disease, ulcerative colitis, or Crohn disease) [26–30]. Here, the BTNL2 rs2076530 splice variant could not distinguish sporadic from familial forms and we still have to understand pathways through which the truncated form of BTNL2 may be involved in the pathogenesis of sarcoidosis.

Sarcoidosis seems to be a multifactorial disease that involves various genetic and environmental pathogenic factors. To date, strong roles are suggested for different SNPs in various genes mostly identified by association studies: HLA-DP subregion, BTNL2, Annexin A11, and, more recently Toll-like receptors, coiled-coil domain-containing protein 88B, Ataxin-2/SH2B adapter protein 3, interleukin IL12B, and Beta-mannosidase/Nuclear factor NF-kappa-B p105 subunit [8, 31–36]. More than ten genes might then be involved in the predisposition to sarcoidosis and next generation sequencing studies will probably provide new candidates for the molecular pathways of granuloma generation. Finally, sarcoidosis might not be a single disease entity but rather a reaction to various triggering events.

Because the present study data were collected from both prevalent and incident cases (to increase the number of familial cases and recoverable DNAs), two biases may be considered. Firstly, some data were missing because of their retrospective collection and others because the 28 centres might not have given the same number of visits or the same delays between visits to all patients. Secondly, ancestry data relied on the ancestors’ birth countries and several patients had ancestors from distinct continents. However, BTNL2 polymorphism was shown linked to sarcoidosis regardless of ethnicity in Caucasian, Afro-American, and Japanese patients [37]. Lastly, we did not use the clinical outcome status (COS) of the WASOG Task Force [7] because the COS became available four years after the start of the present study.

## Conclusions

This study is an original comparison of sarcoidosis phenotypes and genotypes between familial and sporadic cases in a very large cohort. Despite a significant difference in BTNL2 polymorphism between sarcoid patients and controls, the lack of correlations between polymorphism, inheritance, clinical phenotypes, and outcome argues against the consideration of this single genetic difference as a practical marker for patient classification or optimized individual patient management.

## Abbreviations

BTNL2: Butyrophilin-like 2 (gene)
CI: Confidence interval
FEV1: Forced expiratory volume in one second
FVC: Forced vital capacity
OR: Odds ratio
SARCFAM: Sarcoïdose Familiale (study)
SCAC: Sarcoid Clinical Activity Classification
SNP: Single-nucleotide polymorphism
TLC: Total lung capacity
WASOG: World Association for Sarcoidosis and Other Granulomatous Disorders

## References

[1] Valeyre D, Prasse A, Nunes H, Uzunhan Y, Brillet PY, Müller-Quernheim J (2014). Sarcoidosis. Lancet.

[2] Lin Y, Wei J, Fan L, Cheng D (2015). BTNL2 gene polymorphism and sarcoidosis susceptibility: a meta-analysis. PLoS ONE.

[3] Scadding JG (1961). Prognosis of intrathoracic sarcoidosis in England. A review of 136 cases after five years’ observation. BMJ.

[4] Wasfi YS, Rose CS, Murphy JR (2006). A new tool to assess sarcoidosis severity. Chest.

[5] Rodrigues SC, Rocha NA, Lima MS, Arakaki JS, Coletta EN, Ferreira RG (2011). Factor analysis of sarcoidosis phenotypes at two referral centers in Brazil. Sarcoidosis Vasc Diffuse Lung Dis.

[6] Prasse A, Katic C, Germann M, Buchwald A, Zissel G, Müller-Quernheim J (2008). Phenotyping sarcoidosis from a pulmonary perspective. Am J Respir Crit Care Med.

[7] Baughman RP, Nagai S, Balter M (2011). Defining the clinical outcome status (COS) in sarcoidosis: results of WASOG Task Force. Sarcoidosis Vasc Diffuse Lung Dis.

[8] Valentonyte R, Hampe J, Huse K, Rosenstiel P, Albrecht M, Stenzel A (2005). Sarcoidosis is associated with a truncating splice site mutation in BTNL2. Nat Genet.

[9] Rybicki BA, Walewski JL, Maliarik MJ, Kian H, Iannuzzi MC, ACCESS Research Group (2005). The BTNL2 gene and sarcoidosis susceptibility in African and Whites. Am J Human Genet.

[10] Li Y, Wollnik B, Pabst S, Lennarz M, Rohmann E, Gillissen A (2006). BTNL2 gene variant and sarcoidosis. Thorax.

[11] American Thoracic Society, European Respiratory Society, World association of Sarcoidosis and Other Granulomatous Disorders (1999). Statement on sarcoidosis: joint statement of the American Thoracic Society (ATS), European Respiratory Society (ERS), World association of Sarcoidosis and Other Granulomatous Disorders (WASOG) adopted by the ATS Board of Directors and by the ERS Executive Committee, February 1999. Am J Respir Crit Care Med.

[12] Vazquez AI, Bates DM, Rosa GJ, Gianola D, Weigel KA (2010). Technical note: An R package for fitting generalized linear mixed models in animal breeding. J Anim Sci.

[13] Shaffer JP (1995). Multiple Hypothesis Testing. Ann Rev Psychol.

[14] Rybicki BA, Iannuzzi MC, Frederick MM, Thompson BW, Rossman MD, Bresnitz EA (2001). ACCESS Research Group. Familial aggregation of sarcoidosis. A case–control etiologic study of sarcoidosis (ACCESS). Am J Respir Crit Care Med.

[15] Rossman MD, Kreider ME (2007). Lesson learned from ACCESS. Proc Am Thorac Soc.

[16] Sharma OP, Neville E, Walker AN, James DG (1976). Familial sarcoidosis: a possible genetic influence. Ann N Y Acad Sci.

[17] Neville E, Walker AN, James DG (1983). Prognostic factors predicting the outcome of sarcoidosis: an analysis of 818 patients. Q J Med.

[18] Brennan NJ, Crean P, Long JP, Fitzgerald MX (1984). High prevalence of familial sarcoidosis in an Irish population. Thorax.

[19] Nassif X, Valeyre D, Loiseau A, Battesti JP (1985). Familial sarcoidosis. Apropos of 22 families. Ann Med Interne (Paris).

[20] Delaveri A, Rapti A, Poulou M, Fylaktou E, Tsipi M, Roussos C (2014). BTNL2 gene SNPs as a contributing factor to sarcoidosis pathogenesis in a cohort of Greek patients. Meta Gene.

[21] Wijnen PA, Voorter CE, Nelemans PJ, Verschakelen JA, Bekers O, Drent M (2011). Butyrophilin-like 2 in pulmonary sarcoidosis: a factor for susceptibility and progression. Hum Immunol.

[22] Wennerström A, Pietinalho A, Lasota J, Salli K, Surakka I, Seppänen M (2013). Major histocompatibility complex class II and BTNL2 associations in sarcoidosis. Eur Respir J.

[23] Grunewald J, Brynedal B, Darlington P, Nisell M, Cederlund K, Hillert J (2010). Different HLA-DRB1 allele distributions in distinct clinical subgroups of sarcoidosis patients. Respir Res.

[24] Sato H, Grutters JC, Pantelidis P, Mizzon AN, Ahmad T, Van Houte AJ (2002). HLA-DQB1 0201. A marker for good prognosis in British and Dutch patients with sarcoidosis. Am J Respir Cell Mol Biol.

[25] Sato H, Woodhead FA, Ahmad T, Grutters JC, Spagnolo P, van den Bosch JM (2010). Sarcoidosis HLA class II genotyping distinguishes differences of clinical phenotype across ethnic groups. Hum Mol Genet.

[26] Johnson CM, Traherne JA, Jamieson SE, Tremelling M, Bingham S, Parkes M (2007). Analysis of the BTNL2 truncating splice siter mutation in tuberculosis, leprosy and Crohn’s disease. Tissue Antigens.

[27] Mochida A, Kinouchi Y, Negoro K, Takahashi S, Takagi S, Nomura E (2007). Butyrophilin-like 2 gene is associated with ulcerative colitis in the Japanese under strong linkage disequilibrium with HLA-DRB1 1502. Tissue Antigens.

[28] Sato H, Spagnolo P, Silveira L, Welsh KI, du Bois RM, Newman LS (2007). BTNL2 allele associations with chronic beryllium disease in HLA-DPB1 GLU69-negative individuals. Tissue Antigens.

[29] Ali S, Chopra R, Aggarwal S, Srivastava AK, Kalaiarasan P, Malhotra D (2012). Association of variants in, BAT1-LTA-TNF-BTNL2 genes within 6p21-3 region show graded risk to leprosy in unrelated cohorts of Indian population. Human Genet.

[30] Möller M, Kwiatkowski R, Nebel A, van Helden PD, Hoal EG, Schreiber S (2007). Allelic variation in BTNL2 and susceptibility to tuberculosis in a South African population. Microbes Infect.

[31] Schürmann M, Bein G, Kirsten D, Schlaak M, Müller-Quernheim J, Schwinger E (1998). HLA-DQB1 and HLA-DPB1 genotypes in familial sarcoidosis. Respir Med.

[32] Hofmann S, Franke A, Fisher A, Jacobs G, Nothnagel M, Gaede KI (2008). Genome-wide association study identifies ANXA11 as a new susceptibility locus for sarcoidosis. Nat Genet.

[33] Veltkamp M, van Moorsel CH, Rijkers GT, Ruven HJ, Grutters JC (2012). Genetic variation in the Toll-like receptor gene cluster (TLR10-TLR1-TLR6) influences disease course in sarcoidosis. Tissue Antigens.

[34] Fischer A, Schmid B, Ellinghaus D, Nothnagel M, Gaede KI, Schürmann M (2012). A novel sarcoidosis risk locus for Europeans on chromosome 11q13.1. Am J Respir Crit Care Med.

[35] Fischer A, Ellinghaus D, Nutsua M, Hofmann S, Montgomery CG, Iannuzzi MC (2015). Identification of immune-relevant factors conferring sarcoidosis genetic risk. Am J Respir Crit Care Med.

[36] Smith G, Brownell I, Sanchez M, Prystowsky S (2008). Advances in the genetics of sarcoidosis. Clin Genet.

[37] Suzuki H, Ota M, Meguro A, Katsuyama Y, Kawagoe T, Ishihara M (2012). Genetic characterization and susceptibility for sarcoidosis in Japanese patients: risk factors of BTNL2 gene polymorphisms and HLA class II alleles. Invest Ophtalmo Vis Sci.

